# The Human Respiratory System and its Microbiome at a Glimpse

**DOI:** 10.3390/biology9100318

**Published:** 2020-10-01

**Authors:** Luigi Santacroce, Ioannis Alexandros Charitos, Andrea Ballini, Francesco Inchingolo, Paolo Luperto, Emanuele De Nitto, Skender Topi

**Affiliations:** 1 Ionian Department, Microbiology and Virology Laboratory, University of Bari “Aldo Moro”, Piazza G. Cesare 11, 70124 Bari, Italy; luigi.santacroce@uniba.it; 2 Department of Clinical Disciplines, University of Elbasan, Rruga Ismail Zyma, 3001 Elbasan, Albania; skender.topi@uniel.edu.al; 3 Poison Center, OO. RR. University Hospital of Foggia, Viale Luigi Pinto 1, 71122 Foggia, Italy; 4 Department of Biosciences, Biotechnologies and Biopharmaceutics, University of Bari “Aldo Moro”, Via Orabona 4, 70125 Bari, Italy; 5 Department of Precision Medicine, University of Campania “Luigi Vanvitelli”, Vico L. De Crecchio 7, 80138 Naples, Italy; 6 Department of Interdisciplinary Medicine, University of Bari “Aldo Moro”, Piazza G. Cesare 11, 70124 Bari, Italy; francesco.inchingolo@uniba.it; 7 ENT Service, Brindisi Local Health Agency, Via Dalmazia 3, 72100 Brindisi, Italy; p.luperto@libero.it; 8 Department of Basic Medical Sciences, Neurosciences and Sense Organs, University of Bari “Aldo Moro”, Piazza G. Cesare 11, 70124 Bari, Italy; emanuele.denitto@uniba.it

**Keywords:** human microbiome, respiratory microbiome, clinical microbiology, immune modulation, dysbiosis, respiratory diseases, translational research, asthma, SARS-CoV-2

## Abstract

**Simple Summary:**

New data in the current scientific literature show that the composition of the respiratory system microbiome differs in health and disease conditions and that the microbial community as a collective entity can contribute to the pathophysiological processes associated with chronic airway disease. The respiratory microbiome is less studied than that of other areas, but it is believed to contribute to the host’s local immune education and to the development of respiratory diseases, including allergies, asthma and others. In this review, was highlighted the current clinical microbiology knowledge about the microbiota and the various lung diseases relationships, previously only indirectly related to microbial pathogenesis, and the microbiota–pathogenesis relationship of lung infection, among the main causes of diseases, in order to prevent and help in a targeted treatment of various lung diseases.

**Abstract:**

The recent COVID-19 pandemic promoted efforts to better understand the organization of the respiratory microbiome and its evolution from birth to adulthood and how it interacts with external pathogens and the host immune system. This review aims to deepen understanding of the essential physiological functions of the resident microbiome of the respiratory system on human health and diseases. First, the general characteristics of the normal microbiota in the different anatomical sites of the airways have been reported in relation to some factors such as the effect of age, diet and others on its composition and stability. Second, we analyze in detail the functions and composition and the correct functionality of the microbiome in the light of current knowledge. Several studies suggest the importance of preserving the micro-ecosystem of commensal, symbiotic and pathogenic microbes of the respiratory system, and, more recently, its relationship with the intestinal microbiome, and how it also leads to the maintenance of human health, has become better understood.

## 1. Introduction

The human body is exposed to millions of microorganisms through simple daily functions, such as breathing, causing a direct contact. There are areas with a permanent presence of germs (the oral cavity, the respiratory tract, gastrointestinal tract, skin, etc.) but also germfree areas (blood, muscles, brain, etc.) [1]. In 2001, Nobel Prize winner Joshua Lederberg introduced the new scientific term “microbiome”, which refers to the set of genes of all microorganisms that live in and within the human body (skin, mouth, intestine, etc.), that is, in the their genome, and thus, an microecosystem of commensal, symbiotic and pathogenic microbes. This understanding of the important relationship that develops between microbes and the human body was the reason for further investigation into the role of the microbiome in the health and disease of the human body [1,2]. Therefore, in 2008, an international program was established by the United States National Institutes of Health (NIH), the “Human Microbiome Project”, or the scientific program for the microbiome. A population of these “good” germs gradually colonizes the human body from birth, externally and internally, and forms the classic so-called “normal microbial flora”, currently reported as microbiota [2,3]. The total number of these microbes, and in particular of microbial cells, is around 10^14^, which is ten times more than human cells and colonized by six phyla: *Actinobacteria*, *Bacteroidetes*, *Cyanobacteria, Firmicutes, Fusobacteria* and *Proteobacteria* [1]. The microbiome of each human being differs in terms of the types of microorganisms, the population of each species and the relationship between them. Thus, human health and diseases are linked not only to the expression of the genes they carry, but also to the expression of the genes of the microbes they host [1,4,5,6,7].

In this study, we researched the current clinical microbiology knowledge about the microbiota and the various lung diseases relationships, previously only indirectly related to microbial pathogenesis, and the microbiota–pathogenesis relationship of lung infection, among the main causes of diseases, in order to prevent and help in a targeted treatment of various lung diseases. In fact, the respiratory system microbiome (Nasopharyngeal, Tracheobronchial, and Pulmonary districts) is characterized by the presence of bacteria, fungi and viruses that in normal healthy subjects presents a low density and a high diversity of bacterial colonies, unlike what is observed in pathological conditions (infections, asthma, chronic obstructive pulmonary disease (COPD), cystic fibrosis, etc.) in which an increase in certain germs and the reduction in others occurs. The lungs are directly exposed to the external environment, and exposure to microorganisms, allergens, pollutants and other things, can alter the composition of the microbiota. Respiratory diseases (congenital or not) can then alter the balance of immigration and elimination since the inhaled germs find a more favorable habitat in the lung for their development but also a reduced effectiveness of the factors that help their elimination [4,5]. Pulmonary diseases can then cause dysbiosis in the microbiota (in which an increase in certain germs and a reduction in others are observed) and consequently an ineffectiveness of the mechanisms of elimination of germs (cough, muco-ciliary clearance). Furthermore, they can cause alterations of the structure of the airways (bronchi, bronchioles, alveoli) and thus changes in the viscosity of the mucus, in the pH, and in O_2_ tension, hence the alteration of ventilation and perfusion in the alveolar–capillary membrane. These modifications in turn facilitate the formation of niches that favor the growth and increase of common commensal bacteria. In addition, the interaction between the intestine and the lungs plays an important role in pulmonary microbiome eubiosis and immunomodulation [8,9,10]. It then becomes important to investigate the role of: (a) pathogenic microbes that cause or contribute to disease development and/or progression, (b) commensal microbes that do not cause disease, (c) neutral or beneficial host interaction bacterial microbiota studies, (d) the recent reassessment of viruses and fungi [11,12,13].

## 2. The Upper Respiratory Tract Microbiome (Airways Colonization and Evolution during Life)

The upper respiratory system is anatomically related to an interconnected cavity system that includes the nostrils, rhino-pharynx, and oropharynx, and communicates with the larynx and the middle cavity of the ear through the Eustachian tube. The mucous surfaces of these areas are colonized by a wide range of bacteria belonging to the genera *Firmicutes, Actinobacteria, Bacteroidetes, Proteobacteria* and *Fusobacteria* [1,4,7]. The main functions of the upper respiratory system are to filter, heat and humidify the air that passes through it before it reaches the lungs. The individual anatomical regions have their own special characteristics (humidity, temperature, relative oxygen concentration, type of epithelial cells, etc.) They create their microenvironment with the result that there are differences in the microbiome at lower taxonomic levels of the microorganisms that colonize each region. The composition of the microbial community is influenced by environmental factors and the interactions of microbes and their host immune system. Normal microbiota prevents the formation of pathogenic microorganisms. The pathogen competes for attachment sites, for nutrients and is difficult to install to multiply and cause disease. Competition between germs is also a factor shaping the microbiome [8,9,10,11]. Colonization of the upper respiratory tract begins at birth and the association of the original microbiome with health established throughout a person’s life with three main modalities: (a) method of delivery (caesarean or normal); (b) environment (habitat, diet etc.); and (c) antibiotics. The rhino-pharynx and oropharynx microbiome at birth and infancy affected by the individual’s environmental exposures, including breastfeeding. Environment habitat has an important role in the develop of the immune system of the lung and the exposure in the first months of life to certain bacteria directs the immunological activity of the child. Recent studies have highlighted how the integrity of the composition and correct maturation of the microbiota in the first period of life can influence the prevention of certain lung diseases or can, in the event of its alteration, cause different pathological states [1,14,15,16,17]. In the first period of life, the signals encoded by the microbiome, both intestinal and pulmonary (Gut/Lung axis), are essential to target the maturation of the cells of the epithelium of airways and affect the maturation of the immune system. However, when bacterial colonization took place in the first year of their age, there seemed to be no association with hissing (wheezing) breath. At 1.5 months of age, five groups predominate: *Streptococcus*, *Moraxella*, *Staphylococcus*, *Corynebacterium* or *Corynebacterium/Dolosigranulum* (colonization by *S. pneumoniae* frequently occurs in children and is asymptomatic and this means that colonization is short and progresses towards infection). The rhino-pharyngeal microbiota of the elderly seems to undergo profound changes. However, it is unclear how these changes affect the composition and maintenance of the microbiome in the upper respiratory tract [1,10,18,19,20]. 

### 2.1. Nostrils

The area of the nostril does not differ much in the characteristics between children and adults. However, children have an abundance of *Streptococcaceae*, *Moraxellaceae* and *Neisseriaceae* families, which have not been confirmed as a mere dispersion from the nearby rhino-pharynx where they predominate or are the result of substantial differentiation of the microbial community from that of adults. In addition, age-related local immunity affects the composition of the microbiome. Antimicrobial peptides, local immune cells such as neutrophils and Natural killer cells (NK), are the first defense against microorganisms [1,5,10,11]. The nostril microbiome is enriched mainly with members of the genera *Actinobacteria (Corynebacterium* and *Propionibacterium spp*.) and *Firmicutes* (*Streptococcus* species in children and *Staphylococcus* species in adults). In small amounts, there are anaerobes belonging to the genus *Bacteroidetes*. As for *Proteobacteria*, their number varies greatly with studies that report a high abundance of *Moraxellaceae* in children and members of the class *Gammaproteobacteria* in healthy adults [1,8,18]. The epithelium of the nostrils contains glands that secrete sebum, and this is related to its selective enrichment with lipophilic bacteria such as *Propionibacterium* spp., able to metabolize sebum lipids into short-chain fatty acids. The pH decreases and promotes the growth of *Corynebacterium* and *Staphylococcus coagulase*. Moreover, the nostrils are rich in oxygen and humidity of contributes to the growth of *Staphylococcus aureus* and *Corynebacterium*. Therefore, the simultaneous presence of *Propionibacterium* and *Staphylococcus* spp. can be supported by different characteristics of the local environment. Their coexistence can be supported by the production of *coproporphyrin* III by *Propionibacterium* spp., which promotes the formation of the *Staphylococcus aureus* biofilm [4,10,18].

### 2.2. Rhinopharynx 

The rhino-pharyngeal microbiome ripples from the beginning of life onwards. Initially, there is a predominance of species belonging to the genera *Moraxella, Corynebacterium, Dolosigranulum, Streptococcus or Staphylococcus spp*. and possibly related to the mode of delivery (during caesarean section microbiome: *Staphylococcus and Corynebacterium spp.* and the microbiome during vaginal delivery: *Staphylococcus, Streptococcus* and *Dolosigranulum spp*.), and the type of diet. Later, in adults, there is a clear absence of *Moraxella*. The species that colonize the rhino-pharynx overlap with species from nearby anatomical areas such as the oropharynx (*Streptococcus* spp) and the anterior roots (mainly aerobic Gram-positive such as *Staphylococcus, Dolosigranulum, Corynebacterium*) [7,10,11]. The Gram-negative anaerobes are the same found mainly in the oropharynx and oral cavity, such as *Prevotella* and *Veillonella* spp mainly in the rhino-pharynx of young children. The rhino-pharyngeal microbiome plays an important and beneficial role in maintaining the balance of related species, preventing the growth of pathogens and host immunity [18,19,20,21].

### 2.3. Oropharynx

**Figure 1:**
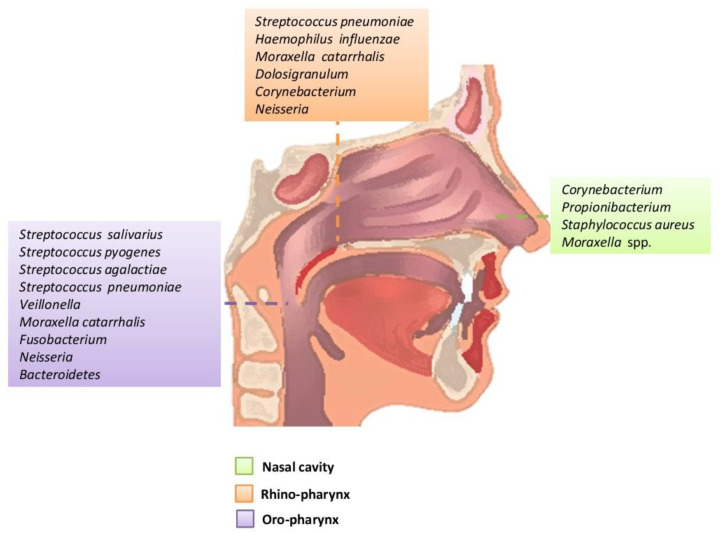
Main bacteria genera and microbiome species of an adult in the upper respiratory tract.

The oropharynx, due to its location, is exposed to a wide variety of microorganisms of endogenous and exogenous origin. It is anatomically linked to the oral cavity, the rhino-pharynx, the larynx, the lower respiratory tract, and the gastrointestinal tract. The germs of the pharyngeal oral community can be transmitted to the lower respiratory tract in healthy and inhaled patients. In the healthy adult, the rhino-pharynx is shown to colonize the genera *Streptococcus, Haemophilus* and *Neisseria* spp. and Gram-negative anaerobic species *Veillonella, Prevotella, Leptotrichia* and *Fusobacterium.* Several pathogens of the genus *Streptococcus* are found in the pharynx, such as *S. pneumoniae*, *S. pyogenes* (which can cause serious disorders that are not limited to the pharynx, and can also cause septic shock) and *S. agalactiae* (Figure 1) [1,10,18,22,23].

## 3. The Lower Respiratory Tract Microbiome (Airway Colonization and Evolution during Life)

In the first period of life, the signals encoded by both the intestinal and pulmonary microbiome are essential to direct the maturation of the cells of the epithelium of the airways and influence the maturation of the immune system. Since 2010, numerous studies have shown, thanks to new DNA and RNA investigation techniques, that the lung microbiome of healthy subjects is composed of bacteria, viruses, Bacteriophages, and fungi such as *Aspergillus, Cladosporium, Eurotium, Penicillum,* and others. Bacterial phylum composition, in order of population number, is as follows: *Bacteroidetes, Firmicutes, Proteobacteria, Fusobacteria,* and *Actinobacteria.* At the gender level, there are *Veillonella, Prevotella, Fusobacteria, Streptococcus* and others (less presence of potential pathogens such as *Haemophilus*) (Table 1) [1,24,25].

The lung microbiome is determined in the first years of life and changes with age, diet, living environment and the use of antibiotics. The lungs do not have a similar microbiome in all tracts (bronchi, bronchioles, alveoli) and, therefore, the pulmonary composition depends on a multitude of factors, in particular: (a) microbial immigration (micro-aspiration, inhalation of microorganisms, direct mucous dispersion), (b) microbial elimination (cough, muco-ciliary clearance, innate and adaptive immunity) and (c) local growth conditions (nutritional availability, temperature, partial O_2_ tension, local microbial competition, concentration and activity of inflammatory cells). The reduction in the microbial elimination capacity both increases regional growth conditions and creates dysbiosis, and therefore leads to a high risk of lung disease [24,26,27].

## 4. Pathogenesis of Respiratory Disorders

It is recognized that people have developed relationships with their symbiotic bacteria, which are essential for good health. However, local changes that alter this symbiosis, creating the condition of dysbiosis, lead to diseases, such as respiratory infections, allergies and asthma, which are due in part to the first colonization, influenced mainly by the modality of delivery (caesarean section, normal) but also by breastfeeding [3,12].

**Figure 2:**
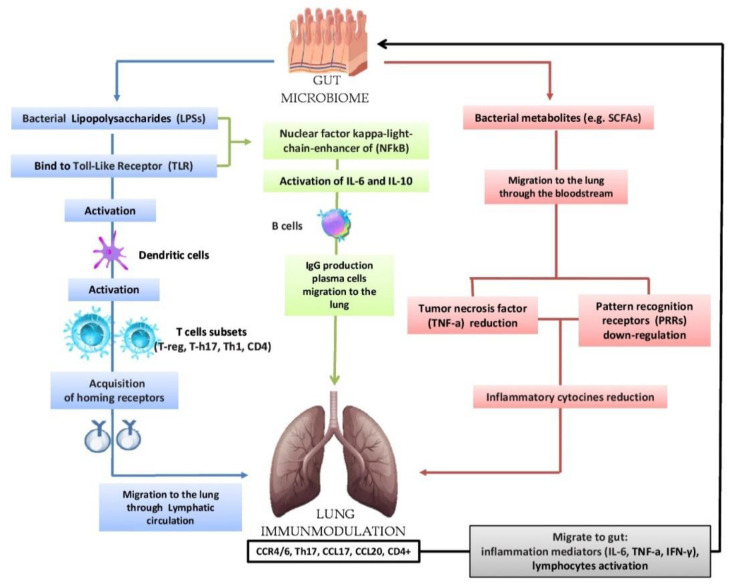
Bidirectional hypothesis that the intestinal microbiota can modulate the immunological activity of the lung: lipopolysaccharides (LPSs) are able to bind to the Toll-Like Receptor (TLR) present on the intestinal mucosa causing the activation of dendritic cells that favor the activation of various T cells (T-reg, T-h17, Th-1, CD4); subsequently, there is the acquisition of horning receptors (CCR6, CCR9, CCR4, a4b7) and migration to the lung through the circulatory lymphatic flow (CCR4/6Treg, CCR4/6Th17, CCR4/6Th1, CCR4/6CD4 +). It also activates (IL-18, INF-y, TNF-a, TGF-b, IL-4, IL-1), which will pass into the circulation (INF-y, TNF-a, IL-6). The nuclear factor kappa-light chain enhancer of (NFkB) activates IL-6, IL-10 and the production of IgA and IgG plasma cell b cells and the migration of IgG to the lung. In the lung, we have the increase in CCL17, CCL20 and the presence of CCR4/6, CD4 +, CCR4/6 and Th17. The migration of bacterial metabolites (e.g., short-chain fatty acids (SCFAs) to the lung through the bloodstream results in the down-regulation of pattern recognition receptors (PRR) with a consequent reduction in the production of inflammatory cytokines (IL-1, IL-12, IL-18), tumor necrosis factor alpha (TNF-α), interferon gamma (IFNγ) and granulocyte–macrophage colony stimulating factor (GM-CSF). In turn, the lungs send inflammation mediators and lymphocytes to the gut in case of disease.

Nasopharyngeal infections are quite common worldwide and show high morbidity. In contrast, lower respiratory tract infections are relatively few but have a high mortality rate. Nasal and oral cavities are unique and determine the bacteria that will grow and evolve. As it gets older, the microbiome of the mouth and pharynx becomes quite similar. Depending on the area where pathogenic bacteria grow, they can cause local dysfunction (pharyngitis) or diffuse disease (pneumonia) [28,29,30]. The rhino-pharynx has many potential pathogenic bacteria such as *S. pneumoniae*, *H. influenzae* and *S. aureus*, and these species are considered to be part of normal microbiota. On the other hand, the rhino-pharynx is also a reservoir for germs associated with acute respiratory infection. The diffusion of the *S. pneumoniae* from the rhino-pharynx can lead to pneumonia, meningitis, or sepsis. In young adults, colonization is rarer and shorter in duration due to strong immune response and therefore the disease is rare unless there are other reasons or a flu-like infection [14,28,29]. In the elderly, transmission rates are low but there is a high incidence of pneumonia. There are studies that suggest that the changes that occur in the microbiome of the elderly contribute to increasing susceptibility to respiratory infections. It is unknown whether specific bacterial species or the general dynamics of the bacterial community make the elderly susceptible to respiratory infections. Age-related immune system changes contribute to the increased incidence of respiratory infections [28,30,31]. The sinuses are cavities around the nose where inflammations may occur such as acute or recurrent sinusitis. Sinusitis can affect both the ethmoidal, sphenoidal, and frontal sinuses. The sinuses are usually an individual microbiome area but also can not be contaminated by nasal and oral cavity microbiota, usually under accumulation of secretions with the main bacteria involved, *Pneumococcus*, *Haemophilus* and *Streptococcus.* Finally, rhinitis, pharyngitis and tonsillitis caused by a heterogeneous group that includes viruses and bacteria (*Hemolytic β streptococcus* is the most common cause and covers 15% of cases) [32,33,34,35,36,37]. Quite often, however, these infections are due to rhinovirus, adenovirus, infectious mononucleosis virus, coronavirus (such as the new pandemic SARS-CoV-2). SARS-CoV-2 in the upper airway tract can lead to the complication of hyposmia/anosmia and hypogeusia/ageusia interacting directly with neural tissues or via the immune system. In fact, according to some hypotheses, such symptoms are linked to the neuronal cells’ impairment or to ischemic harm of the central nervous system, but also to an increase in Interleukin-6 [38,39,40,41,42]. Otitis is an acute infection of the middle ear and occurs most often in children and it is mainly due to *Pneumococcus*, *Haemophilus* and *Staphylococcus* [5,22,43]. Several factors can increase or decrease the risk of appearance of a lung disease. The composition of the pulmonary microbiota depends on three main factors: (a) microbial immigration (micro-aspiration, inhalation of microorganisms, direct mucosal dispersion), (b) microbial elimination capacity (cough, muco-ciliary clearance, innate and adaptive immunity), (c) regional growth conditions (nutritional availability, temperature, O_2_ tension, local microbial competition, concentration and activity of inflammatory cells). The reduction in the microbial elimination capacity both increases regional growth conditions and creates dysbiosis, and therefore leads to a high risk of lung disease (Figure 2). These modifications facilitate the formation of niches that favor the growth and increase in *Prevotella* and *Veillonella* capable of inducing inflammation in the airways through the production of neutrophils and lymphocytes. They therefore lead to dysbiosis of the microbiota, inflammation, and lung damage. It is assumed that the modified pulmonary microbiota loses its protective capacity and may play a potential role in the pathogenesis of chronic lung diseases, or in asthma, cystic fibrosis, chronic obstructive pulmonary disease, broncho-dysplasia, and idiopathic pulmonary fibrosis [44,45,46,47].

Inflammation of the lungs resulting from infection during childhood is associated with the development of asthma. Thus, microbiota dysbiosis of the airways could be the basis for the susceptibility and progression of chronic lung disease. Most newborns first colonize with *Staphylococcu*s or *Corynebacterium* before stable colonization with *Alloiococcus* or *Moraxella*: the link between bacterial colonization of the airways in children and the onset of asthma later in life is noted. Infants whose pharynx has been colonized by *Streptococcus pneumoniae*, *Haemophilus influenzae* or *Moraxella catarrhalis* since the beginning of their life have increased asthma risk. These same bacteria are constantly associated with the worsening of asthma, such as COPD. 

**Figure 3:**
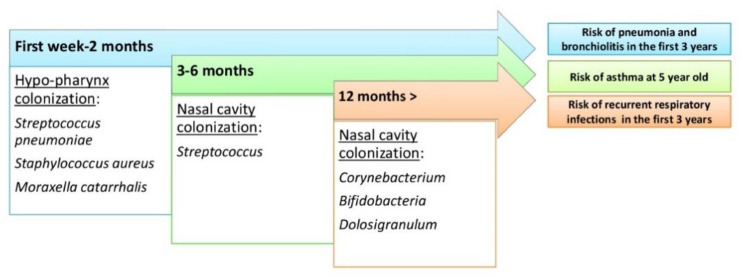
The evolution of the upper respiratory tract microbiome during human life and risk of respiratory tract diseases.

However, exposure to a wider range of germs seems to have a protective effect on the development of asthma in children by activating the innate immune system. This finding will support the hypothesis that asthma caused by a lack of microbial exposure at the beginning of life has consequent effects on the development of the immune system. Epidemiological research has consistently shown that a rich microbial environment in early childhood provides protection against the development of asthma, suggesting the need to understand the extent and nature of the normal microbiota of the airways. Another study showed that two-month age streptococcal colonization was a strong predictor of asthma later in life (Figure 3) [48,49,50,51].

**Figure 4:**
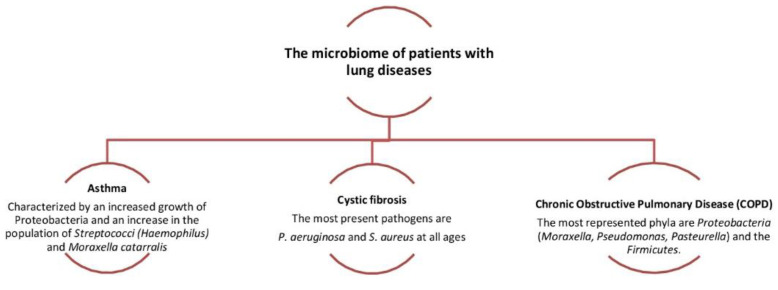
Dysbiosis related to some lung diseases. The relative abundance in *Proteobacteria* is associated with the increased severity of chronic obstructive pulmonary disease (COPD).

Chronic obstructive pulmonary disease is characterized by (a) a persistent inflammation, (b) dysfunction of muco-ciliary activity, and (c) structural and functional alterations affecting bronchi leading to a partially reversible obstruction. The progression of the severity of the damage of the lung structures starts through the frequent exacerbations. Indeed, several studies used molecular analysis of the bacterial gene 16S-rRNA to characterize the synthesis of bacterial communities from adult airways, including patients with asthma and chronic respiratory pneumonia (COPD). Those identified 190 genera, mainly belonging to the genera *Prevotella*, *Streptococcus*, *Staphylococcu*s, *Neisseria*, *Corynebacterium* and *Haemophilus* spp., and nasal cavity samples were highly characterized by *Actinobacteria* (mainly *Corynebacterium* spp.) and *Firmicutes* (mainly *Staphylococcus* spp.) The most common *oropharyngeal region* bacteria are mainly the *Prevotella* spp., and from the left upper lobe samples are mainly *Haemophilus* spp.; the use of antibiotics increased the likelihood of colonization by *Streptococcus, Haemophilus* and *Moraxella*. (Figure 4) [52,53,54,55,56,57,58].

## 5. The Gut-Lung Axis

### 5.1. Biomolecular Mechanisms

The lungs have a different habitat depending on their anatomic components (bronchi, bronchioles, alveoli). In lung diseases such as inflammation, acute respiratory distress syndrome, septic state, etc. the lung microbiota becomes rich in intestinal bacteria, such as *Bacteroidetes* and *Enterobacteriaceae*. This phenomenon is also called “more gut in the lung”. In acute situations, the intestine becomes hyper-impermeable (leaky gut) and bacteria can translocate through the colon wall and reach the lung, influencing inflammation, infection and acute lung damage. Interconnection is particularly important between the lung microbiome and the intestinal microbiome and it known that there is an exchange of immunological information between the two apparatuses and the possibility of influencing the functional behavior of the lung microbiome under certain conditions. It has been hypothesized that the gut microbiome can modulate the immunological activity of the lung by three main modalities: (a) The production of bacterial ligands (lipopolysaccharides), (b) production of bacterial metabolites (e.g., short-chain fatty acids (SCFAs), and (c) migration of immune cells (T-cells to the lung through the lymphatic stream). The complementary Dendritic Cells (DCs) activate T-cell subpopulations in the mesenteric lymph nodes (MLN) and gastro-intestinal lymphatic tissue (GALT) and produce regulatory cytokines. Following airway immune stimulation, T-cells are activated in the GALT and MLN, move to the respiratory epithelium and provide protection and anti-inflammatory action. The SCFAs from the intestine reach the lungs and suppress inflammation [9,12,13].

Several studies have confirmed this theory by noting that the depletion of the intestinal microbiota causes severe pneumonia, which attenuates with the restoration of normal microbiota. It has been noted that strains of *Bifidobacterium*, *Lactobacillus* and *Clostridium* cause an increase in T-reg cells. It has also been noted that an exopolysaccharides (EPSs) produced by *Bifidobacterium longum* appear to be able to repress the inflammatory response of the host by suppressing the production of Th17 in the intestine and lung (Figure 2) [20,59,60,61]. The presence in the intestinal microbiome of high levels of fecal *Clostridium difficilis* at the age of one month is associated with a high risk of eczema and asthma at the age of 6–7 years. The transient reduction during the first 100 days of *Veillonella* and *Faecalibacterium prausnitzii* correlates with an increased risk for asthma, as well as colonization with *Bacteroides fragilis* (Figure 3) [20,62,63].

Finally, in terms of this well-known relationship between microbiome and health, one of the problems that is still being addressed concerns the causality of the microbiome–disease relationship. To address this problem, different animal models are being used (such as with rats or other animals). In fact, in the “gnotobiotic” animal model the animals have a known microbiota, but since in addition to the wide cost of the animals, a certain amount of experience is also required for its use, it is therefore not very widespread. Another one is the “conventionalized” animal model (a gnotobiotic evolution model), it uses animals in which the human intestinal microbiota is inserted to colonize the gastrointestinal tract (the microbiota present in all human tissues is transferred to the animal). Therefore, most human diseases can be investigated on animals, thus monitoring the role of the microbiome in the onset and evolution of the disease itself. The aim of these studies is the possibility of controlling and maneuvering the human microbiome in such a way as to reduce the risk of certain diseases or modify specific metabolic and immunological pathways that are harmful to health [64,65].

### 5.2. Effects of Environmental Factors Influencing the Eubiosis of the Airway’s Microbiota 

The lung microbiota colonizes and is distributed in relation to the anatomical characteristics of the lung, its ventilation function, elimination capacities and environmental conditions such as growth room. Thus, the interconnection between lung microbiota and intestinal microbiota is important, and is validated by the presence of the lung microbiota disease that is also common to intestinal microbiota. 

The environment plays an important role in the development and effectiveness of the immune system present in the upper and lower airways, especially after birth and in the first months of life because they are exposed to certain bacteria. Early exposure to microorganisms is protective against the onset of allergic diseases and asthma [66,67]. A recent study has shown that newborns born from caesarean delivery have reduced colonization by beneficial bacteria such as *Corynebacterium* and *Dolosigranulum*; instead, the breastfeeding is associated with a wide levels those species and that can play a key role in protecting the immune system. At the same time, the use of antibiotics increased the likelihood of colonization by *Streptococcus, H. influenzae* and *Moraxella*. Children colonized in the first month of life by *H. influenzae*, *S. pneumoniae*, and *Moraxella catarrhalis* were more likely to develop hissing breath than those not colonized It has been shown that an inadequate but also altered microbial colonization is a probable cause of dysbiosis with a lack of those important and essential microorganisms for the development and maintenance of the immune system [62,68,69,70]. The inoculation of spore-forming *Clostridium* (IV,XIV) on newborn mice causes a reduction in IgE, unlike what occurs in adult mice, thus reducing the risk of allergy [62]. In particular, numerous studies have confirmed that in certain countryside environments such as farms (breast feeding and unpasteurized milk), presence of animals and exposure to endotoxins favor significant protection in adulthood against the onset of allergic sensitization and asthma [71,72,73]. The presence of large quantities of fatty acids and oligosaccharides in breast milk has a protective effect against the onset of asthma and allergies by modifying the composition of the intestinal microbiota (growth of *Lactobacilli* and *Bifidobacteria*, which play a protective role by promoting Th1/Th2 balance) and thus its immune function through the activation of T-reg cells. On the other hand, a diet rich in fiber increases the number of *Bacteroides* and *Actinobacteria* by reducing *Firmicutes* and *Proteobacteria*. This qualitative and quantitative change in the intestinal microbiota determines a greater production of short-chain fatty acids (acetate, propionate and butyrate), which modulate the immune response in the lungs with the effect of reducing Th-2 and eosinophils [73,74,75,76,77].

These first exposures to the gut microbiome have been linked to the development of diseases and conditions such as obesity, type 1 diabetes, asthma, allergies, idiopathic inflammatory disease and even neurodevelopmental disorders. Thus, the composition of the flora is therefore modified and influenced by age, the state of the immune system and various environmental and other factors (Table 2) [1,12,13,78].

In addition, regarding the current COVID-19 pandemic infection caused by the SARS-CoV-2, gastrointestinal involvement is observed as in the other various respiratory tract infections that can complicate these disorders. In fact, it appears from recent studies that gastrointestinal pathology in patients is more frequent and lasting than in pulmonary pathology. This could be linked to the imbalance between lung and intestinal microbiota, and the severe prognosis can be influenced by bacterial dysbiosis. In fact, this influence on the gut microbiota occurs according to some studies through (a) the primary inflammatory stimuli triggering the release of microbial products and cytokines, increasing systemic inflammation, and (b) the increase in blood proteomic risk score (PRS). Furthermore, fecal samples in patients with high infectivity had a wide number of *Collinsella aerofaciens*, *Collinsella tanakaei*, *Streptococcus infantis, Morganella morganii* in contrast to patients with low or no infectivity, who had a wide number of SCFAs, *Parabacteroides merdae, Bacteroides stercoris*, *Alistipes onderdonkii*, and *Lachnospiraceae bacterium*. Consequently, knowing that the intestinal microbiota influences the action of ACE2, we can hypothesize a close relationship between COVID-19 and the microbiota and this in turn modifies the lung microbiota [79,80,81,82,83].

Thus, the use of specific diets, specific living environments, and supplementation with probiotics can be factors that are protective against the onset and progression of respiratory diseases. It also seems appropriate to deepen the interactions between guest and response in the future immune response to the risk of respiratory diseases (Table 3), in order to seek new therapeutic interventions that can positively modulate this response [83,84,85,86,87,88,89].

## 6. Conclusions

In the postnatal and adult stages, the microbiome influences the reproductive, cognitive, metabolic and immunological aspects of the individual; it is argued that the microbiota is suitable to support some functions in the early stages of life while in the latter it contributes to the death of the host. It has emerged that the microbiome is explicit in relation to the age of the individual, causing an increase in cellular proliferation and producing pro-mutagenic metabolites (for example butyrate); it therefore possesses an oncogenic potential. New data show that the composition of the respiratory system microbiome differs in health and disease conditions and that the microbial community as a collective entity can contribute to the pathophysiological processes associated with chronic airway disease. The respiratory microbiome is less studied than that of other areas, but it is believed to contribute to the host’s local immune education and to the development of respiratory diseases, including allergies, asthma and others. Local pathogenic bacteria are associated with respiratory infections such as *Streptococcus pneumoniae* and *Haemophilus influenzae*. However, these bacteria, along with fungi, viruses and other bacteria, inhabit the upper respiratory system where they create the microbiome of the rhino-pharynx, a complex ecological network. When there is a balance between the members of the respiratory microbiome, its action is beneficial for humans through strengthening the immune system and preventing the creation of pathogens. However, if the microbiome is disturbed, e.g., because of an antibiotic therapy, potential pathogens may proliferate locally or be transmitted to other areas, creating some local or systemic infections such as acute rhinosinusitis, otitis media, pneumonia, sepsis, etc. In conclusion, the analysis of the intestinal/respiratory microbiome in the context of COVID-19, and the general exploration of its modulation through the diet or food supplements (prebiotics, probiotics, foods rich in fiber, etc.) and fecal transplants, deserve further investigation in clinical microbiology and translational research. The use of prebiotics and probiotics to regulate the balance of the intestinal flora could be an effective treatment to reduce the risk of bacterial and viral infections and other diseases of the respiratory system, thus preventing lung damage and increasing global human health. 

## Figures and Tables

**Table 1:** Main bacteria and fungi genera of the lower respiratory tract microbiome of adult people.

Bacteria	Fungi
*Prevotella*	*Aspergillus*
*Sphingomonas*	*Cladosporium*
*Pseudomonas*	*Penicillum*
*Acinetobacter*	*Eurotium*
*Fusobacterium*	*Candida*
*Megasphaera*	*Malassezia*
*Veillonella*	*Neosartorya*
*Staphylococcus*	*Saccharomyces*
*Streptococcus*	

**Table 2:** The main factors that modified microbiome composition.

Factors Influencing the Gut Microbiota	
Intrinsic	Extrinsic
Gastric secretion	Age
Oxigen presence/concentration	Delivery (cesarean section, natural)
Motility	Diet (breast milk, formula milk, pre- and probiotic foods)
Mucus and Gastro-Intestinal (GI) secretions	Environmental stress
Antimicrobiale peptides	Infections
Immunity state	Drugs (Proton pump inhibitors, H2 Blockers, Prokinetics, Antibiotics, Laxatives, Opioids, Nonsteroidal anti-inflammatory drugs: NSAIDs)

**Table 3:** References about the respiratory health risk in early years related to the lung and gut microbiota.

Risk of Respiratory Diseases	
Alteration of Lung Microbiota	Alteration of Gut Microbiota
Bisgaard H. et al. (N Engl J Med 2007): Pneumonia and bronchiolitis in the first 3 years	Abrahamsson T.R. et al. (Clin Exp Allergy. 2014): Asthma at 7 years old
Huang YJ and Boushey HA (Am Thorac Soc. 2014): Pneumonia and bronchiolitis in the first 3 years	Fujimura K.E. et al. (Nat Med 2016): Asthma at 6–7 years old
Teo S.M. et al. (Cell Host Microbe 2015): Asthma at 5 years old	Arrieta MC and Finlay B. (J Infect. 2014): Asthma at 5 years old
Hasegawa K. et al. (Allergy 2017): Recurrent respiratory infection in the first 3 years	Stokholm J. et al. (Nat Commun. 2018): Asthma at age 5 years old

## References

[1] Finlay B.B., Finlay J.M. (2019). The Whole-Body Microbiome: How to Harness Microbes―Inside and Out―for Lifelong Health Hardcover.

[2] Turnbaugh P.J., Ley R.E., Hamady M., Fraser-Liggett C.M., Knight R., Gordon J.I. (2007). The human microbiome project. Nature.

[3] Huttenhower C., Gevers D., Knight R., Abubucker S., Badger J.H., Chinwalla A.T., Creasy H.H., Earl A.M., FitzGerald M.G., Fulton R.S. (2012). The Human Microbiome Project Consortium. Structure function and diversity of the healthy human microbiome. Nature.

[4] Sahin-Yilmaz A., Naclerio R.M. (2011). Anatomy and physiology of the upper airway. Proc. Am. Thorac. Soc..

[5] Rogers G.B., Shaw D., Marsh R.L., Carroll M.P., Serisier D.J., Bruce K.D. (2015). Respiratory microbiota: Addressing clinical questions, informing clinical practice. Thorax.

[6] Mitchell A.B., Oliver B.G., Glanville A.R. (2016). Translational aspects of the human respiratory virome. Am. J. Respir. Crit. Care Med..

[7] Campanella V., Syed J., Santacroce L., Saini R., Ballini A., Inchingolo F. (2018). Oral probiotics influence oral and respiratory tract infections in pediatric population: A randomized double-blinded placebo-controlled pilot study. Eur Rev Med Pharmacol Sci..

[8] Biesbroek G., Tsivtsivadze E., Sanders E.A., Montijn R., Veenhoven R.H., Keijser B.J., Bogaert D. (2014). Early respiratory microbiota composition determines bacterial succession patterns and respiratory health in children. Am. J. Respir. Crit. Care Med..

[9] Huffnagle G., Dickson R., Lukacs N. (2017). The respiratory tract microbiome and lung inflammation: A two-way street. Mucosal Immunol..

[10] Kumpitsch C., Koskinen K., Schöpf V., Moissl-Eichinger C. (2019). The microbiome of the upper respiratory tract in health and disease. BMC Biol..

[11] Schenck L.P., Surette M.G., Bowdish D.M.E. (2016). Composition and immunological significance of the upper respiratory tract microbiota. FEBS Lett..

[12] Ranucci G., Buccigrossi V., de Freitas M.B., Guarino A., Giannattasio A. (2017). Early-Life Intestine Microbiota and Lung Health in Children. J. Immunol. Res..

[13] Zhang D., Li S., Wang N., Tan H.Y., Zhang Z., Feng Y. (2020). The Cross-Talk between Gut Microbiota and Lungs in Common Lung Diseases. Front. Microbiol..

[14] Gallacher D.J., Kotecha S. (2016). Respiratory Microbiome of New-Born Infants. Front. Pediatr..

[15] Cicinelli E., Ballini A., Marinaccio M., Poliseno A., Coscia M.F., Monno R., De Vito D. (2012). Microbiological findings in endometrial specimen: Our experience. Arch. Gynecol. Obstet..

[16] DiGiulio D.B. (2012). Diversity of microbes in amniotic fluid. Semin. Fetal Neonatal Med..

[17] Krzych-Fałta E., Furmańczyk K., Lisiecka-Biełanowicz M., Sybilski A., Tomaszewska A., Raciborski F., Wojas O., Walkiewicz A., Samel-Kowalik P., Samoliński B. (2018). The effect of selected risk factors, including the mode of delivery, on the development of allergic rhinitis and bronchial asthma. Adv. Dermatol. Allergol..

[18] David W., Cleary S.C. (2017). Clarke the nasopharyngeal microbiome Emerging Topics in Life. Sciences.

[19] Bosch A.A.T.M., Levin E., van Houten M.A., Hasrat R., Kalkman G., Biesbroek G., de Steenhuijsen Piters W.A.A., de Groot P.C.M., Pernet P., Keijser B.J.F. (2016). Development of Upper Respiratory Tract Microbiota in Infancy is Affected by Mode of Delivery. EBioMedicine.

[20] Ballini A., Cantore S., Farronato D., Cirulli N., Inchingolo F., Papa F., Malcangi G., Inchingolo A.D., Dipalma G., Sardaro N. (2015). Periodontal disease and bone pathogenesis: The crosstalk between cytokines and porphyromonas gingivalis. J. Biol. Regul. Homeost. Agents.

[21] Gnoni A., De Nitto E., Scacco S., Santacroce L., Palese L.L. (2019). A New Look at the Structures of Old Sepsis Actors by Exploratory Data Analysis Tools. Antibiotics.

[22] Di Serio F., Lovero R., D’Agostino D., Nisi L., Miragliotta G., Contino R., Man A., Ciccone M.M., Santacroce L. (2016). Evaluation of procalcitonin, Vitamin D and C-reactive protein levels in septic patients with positive emocoltures. Our preliminary experience. Acta Med. Mediterr..

[23] Ballini A., Dipalma G., Isacco C.G., Boccellino M., Di Domenico M., Santacroce L., Nguyễn K.C.D., Scacco S., Calvani M., Boddi A. (2020). Oral Microbiota and Immune System Crosstalk: A Translational Research. Biology.

[24] Pulvirenti G., Parisi G.F., Giallongo A., Papale M., Manti S., Savasta S., Licari A., Marseglia G.L., Leonardi S. (2019). Lower Airway Microbiota. Front. Pediatr..

[25] Dickson R.P., Erb-Downward J.R., Freeman C.M., McCloskey L., Falkowski N.R., Huffnagle G.B., Curtis J.L. (2017). Bacterial topography of the healthy human lower respiratory tract. MBio.

[26] West J.B. (1978). Regional differences in the lung. Chest.

[27] Dickson R.P., Erb-Downward J.R., Freeman C.M., McCloskey L., Beck J.M., Huffnagle G.B., Curtis J.L. (2015). Spatial Variation in the Healthy Human Lung Microbiome and the Adapted Island Model of Lung Biogeography. Ann. Am. Thorac. Soc..

[28] Basis C.M., Tang A.L., Young V.B., Pynnonen M.A. (2014). The nasal cavity microbiota of healthy adults. Microbiome.

[29] Huang Y.J., Boushey H.A. (2014). The microbiome and asthma. Ann. Am. Thorac. Soc..

[30] Wouter A.A., de Steenhuijsen P., Huijskens E.G.W., Wyllie A.L., Biesbroek G., van den Bergh M.R., Veenhoven R.H., Wang X., Trzciński K., Bonten M.J. (2016). Dysbiosis of upper respiratory tract microbiota in elderly pneumonia patients. ISME J..

[31] Teo S.M., Mok D., Pham K., Kusel M., Serralha M., Troy N., Holt B.J., Hales B.J., Walker M.L., Hollams E. (2015). The infant nasopharyngeal microbiome impacts severity of lower respiratory infection and risk of asthma development. Cell Host Microbe.

[32] Bokulich N.A., Chung J., Battaglia T., Henderson N., Jay M., Li H., Lieber A., Wu F., Perez-Perez G.I., Chen Y. (2016). Antibiotics, birth mode, and diet shape microbiome maturation during early life. Sci. Transl. Med..

[33] Montoya-Williams D., Lemas D.J., Spiryda L., Patel K., Carney O.O., Neu J., Carson T.L. (2018). The Neonatal Microbiome and Its Partial Role in Mediating the Association between Birth by Cesarean Section and Adverse Pediatric Outcomes. Neonatology.

[34] Giudice G., Cutrignelli D.A., Sportelli P., Limongelli L., Tempesta A., Gioia G.D., Santacroce L., Maiorano E., Favia G. (2016). Rhinocerebral Mucormycosis with Orosinusal Involvement: Diagnostic and Surgical Treatment Guidelines. Endocr. Metab. Immune Disord. Drug Targets.

[35] Lee J.T., Frank D.N., Ramakrishnan V. (2016). Microbiome of the paranasal sinuses: Update and literature review. Am. J. Rhinol. Allergy.

[36] Kellner J.D., Vanderkooi O.G., MacDonald J., Church D.L., Tyrrell G.J., Scheifele D.W. (2009). Changing epidemiology of invasive pneumococcal disease in Canada, 1998–2007: Update from the Calgary-area Streptococcus pneumoniae research (CASPER) study. Clin. Infect. Dis..

[37] Marsland B.J., Gollwitzer E.S. (2014). Host-microorganism interactions in pulmonary diseases. Nat. Rev. Immunol..

[38] Santacroce L., Bottalico L., Charitos I.A. (2020). The Impact of COVID-19 on Italy: A Lesson for the Future. Int. J. Occup. Environ. Med..

[39] Passarelli P.C., Santacroce L., D’Addona A., Garcia-Godoy F. (2020). The Coronavirus Disease-19 Infection and the Oral Mucosa. Open Access Maced. J. Med. Sci..

[40] Passarelli P.C., Passarelli G., Charitos I.A., Rella E., Santacroce L., D’Addona A. (2020). COVID-19 and Oral Diseases: How can we Manage Hospitalized and Quarantined Patients while Reducing Risks?. Electron. J. Gen. Med..

[41] Cazzolla A.P., Lovero R., Lo Muzio L., Testa N.F., Schirinzi A.L., Palmieri G., Pozzessere P., Procacci V., Di Comite M., Ciavarella D. (2020). Taste and smell disorders in COVID-19 patients: Role of Interleukin-6. ACS Chem. Neurosci..

[42] Santacroce L., Charitos I.A., Del Prete R. (2020). COVID-19 in Italy: An Overview from the First Case to Date. Electron. J. Gen. Med..

[43] Johnston J.J., Douglas R. (2018). Adenotonsillar microbiome: An update. Postgrad. Med. J..

[44] Faner R., Sibila O., Agustí A., Bernasconi E., Chalmers J.D., Huffnagle G.B., Manichanh C., Molyneaux P.L., Paredes R., Pérez Brocal V. (2017). The microbiome in respiratory medicine: Current challenges and future perspectives. Eur. Respir. J..

[45] Biswas K., Chang A., Hoggard M., Radcliff F.J., Jiang Y., Taylor M.W., Darveau R., Douglas R.G. (2017). Toll-like receptor activation by sino-nasal mucus in chronic rhinosinusitis. Rhinology.

[46] Dickson R.P., Erb-Downward J.R., Martinez F.J., Huffnagle G.B. (2016). The Microbiome and the Respiratory Tract. Ann. Rev. Physiol..

[47] Yadava K., Pattaroni C., Sichelstiel A.K., Trompette A., Gollwitzer E.S., Salami O., von Garnier C., Nicod L.P., Marsland B.J. (2016). Microbiota Promotes Chronic Pulmonary Inflammation by Enhancing IL-17A and Autoantibodies. Am. J. Respir. Crit. Care Med..

[48] Bisgaard H., Hermansen M.N., Buchvald F., Loland L., Halkjaer L.B., Bønnelykke K., Brasholt M., Heltberg A., Vissing N.H., Thorsen S.V. (2007). Childhood asthma after bacterial colonization of the airway in neonates. N. Engl. J. Med..

[49] Hasegawa K., Mansbach J.M., Ajami N.J., Petrosino J.F., Freishtat R.J., Teach S.J., Piedra P.A., Camargo C.A. (2017). The relationship between nasopharyngeal CCL5 and microbiota on disease severity among infants with bronchiolitis. Allergy.

[50] Wenzel S.E. (2012). Asthma phenotypes: The evolution from clinical to molecular approaches. Nat. Med..

[51] Hilty M., Burke C., Pedro H., Cardenas P., Bush A., Bossley C., Davies J., Ervine A., Poulter L., Pachter L. (2010). Disordered microbial communities in asthmatic airways. PLoS ONE.

[52] Huang Y.J., Nariya S., Harris J.M., Lynch S.V., Choy D.F., Arron J.R., Boushey H. (2015). The airway microbiome in patients with severe asthma: Associations with disease features and severity. J. Allergy Clin. Immunol..

[53] Durack J., Boushey H.A., Huang Y.J. (2018). Incorporating the airway microbiome into asthma phenotyping: Moving toward personalized medicine for noneosinophilic asthma. J. Allergy Clin. Immunol..

[54] Simpson J.L., Daly J., Baines K.J., Yang I.A., Upham J.W., Reynolds P.N., Hodge S., James A.L., Hugenholtz P., Willner D. (2016). Airway dysbiosis: Haemophilus influenzae and Tropheryma in poorly controlled asthma. Eur. Respir. J..

[55] Renwick J., McNally P., John B., DeSantis T., Linnane B., Murphy P. (2014). The microbial community of the cystic fibrosis airway is disrupted in early life. PLoS ONE.

[56] Pragman A.A., Kim H.B., Reilly C.S., Wendt C., Isaacson R.E. (2012). The lung microbiome in moderate and severe chronic obstructive pulmonary disease. PLoS ONE.

[57] Simpson J.L., Baines K.J., Horvat J.C., Essilfie A.T., Brown A.C., Tooze M., McDonald V.M., Gibson P.G., Hansbro P.M. (2016). COPD is characterized by increased detection of Haemophilus influenzae, Streptococcus pneumoniae and a deficiency of Bacillus species. Respirology.

[58] Mosca A., Carucci A., Santacroce L., Schettini F., De Mattia D., Miragliotta G. (2003). Streptococcus pneumoniae nasopharyngeal colonization in young healthy children: Rate of carriage, serotype distribution, and antibiotic resistance. New Microbiol..

[59] Basis C.M., Erb-Downward J.R., Dickson R.P., Freeman C.M., Schmidt T.M., Young V.B., Beck J.M., Curtis J.L., Huffnagle G.B. (2015). Analysis of the upper respiratory tract microbiotas as the source of the lung and gastric microbiotas in healthy individuals. MBio.

[60] Samuelson D.R., Welsh D.A., Shellito J.E. (2015). Regulation of lung immunity and host defense by the intestinal microbiota. Front. Microbiol..

[61] Bottalico L., Castellaneta F., Charitos I.A. (2020). From Hydrotherapy to the Discovery of The Gut Microbiota: The Historical Gastrointestinal Health Concept. Pharmacophore.

[62] Atarashi K., Tanoue T., Shima T., Imaoka A., Kuwahara T., Momose Y., Cheng G., Yamasaki S., Saito T., Ohba Y. (2011). Induction of colonic regulatory T cells by indigenous Clostridium species. Science.

[63] Gozdz J., Ober C., Vercelli D. (2016). Innate Immunity and Asthma Risk. N. Engl. J. Med..

[64] Oddy W.H. (2017). Breastfeeding, Childhood asthma, and allergic disease. Ann. Nutr. Metab..

[65] El Aidy S., Hooiveld G., Tremaroli V., Bäckhed F., Kleerebezem M. (2013). The gut microbiota and mucosal homeostasis: Colonized at birth or at adulthood, does it matter?. Gut Microbes.

[66] Martín R., Bermúdez-Humarán L.G., Langella P. (2016). Gnotobiotic Rodents: An In Vivo Model for the Study of Microbe-Microbe Interactions. Front. Microbiol..

[67] Ballini A., Santacroce L., Cantore S., Bottalico L., Dipalma G., Topi S., Saini R., De Vito D., Inchingolo F. (2019). Probiotics efficacy on oxidative stress values in inflammatory bowel disease: A randomized double-blinded placebo-controlled pilot study. Endocr. Metab. Immune. Disord. Drug Targets.

[68] Arrieta M.C., Finlay B. (2014). The intestinal microbiota and allergic asthma. J. Infect..

[69] Abrahamsson T.R., Jakobsson H.E., Andersson A.F., Björkstén B., Engstrand L., Jenmalm M.C. (2014). Low gut microbiota diversity in early infancy precedes asthma at school age. Clin. Exp. Allergy..

[70] Fujimura K.E., Sitarik A.R., Havstad S., Lin D.L., Levan S., Fadrosh D., Panzer A.R., LaMere B., Rackaityte E., Lukacs N.W. (2016). Neonatal gut microbiota associates with childhood multisensitized atopy and T cell differentiation. Nat. Med..

[71] Schuijs M.J., Willart M.A., Vergote K., Gras D., Deswarte K., Ege M.J., Madeira F.B., Beyaert R., van Loo G., Bracher F. (2015). Farm dust and endotoxin protect against allergy through A20 induction in lung epithelial cells. Science.

[72] Lovreglio P., Bukvic N., Fustinoni S., Ballini A., Drago I., Foà V., Guanti G., Soleo L. (2006). Lack of genotoxic effect in workers exposed to very low doses of 1,3-butadiene. Arch. Toxicol..

[73] Loss G., Depner M., Ulfman L.H., van Neerven R.J., Hose A.J., Genuneit J., Karvonen A.M., Hyvärinen A., Kaulek V., Roduit C. (2015). PASTURE study group. Consumption of unprocessed cow’s milk protects infants from common respiratory infections. J. Allergy Clin. Immunol..

[74] Dickson R.P., Erb-Downward J.R., Huffnagle G.B. (2015). Homeostasis and its disruption in the lung microbiome. Am. J. Physiol. Lung Cell Mol. Physiol..

[75] Toscano M., De Grandi R., Grossi E., Drago L. (2017). Role of the Human Breast Milk-Associated Microbiota on the Newborns’ Immune System: A Mini Review. Front. Microbiol..

[76] Trompette A., Gollwitzer E.S., Yadava K., Sichelstiel A.K., Sprenger N., Ngom-Bru C., Blanchard C., Junt T., Nicod L.P., Harris N.L. (2014). Gut microbiota metabolism of dietary fiber influences allergic airway disease and hematopoiesis. Nat. Med..

[77] Crincoli V., Ballini A., Di Comite M., Tettamanti L., Coscia M.F., Mastrangelo F., De Vito D. (2015). Microbiological investigation of medication-related osteonecrosis of the jaw: Preliminary results. J. Biol. Regul. Homeost. Agents.

[78] Enaud R., Prevel R., Ciarlo E., Beaufils F., Wieërs G., Guery B., Delhaes L. (2020). The Gut-Lung Axis in Health and Respiratory Diseases: A Place for Inter-Organ and Inter-Kingdom Crosstalks. Front. Cell Infect. Microbiol..

[79] Santacroce L. (2020). Letter in response to the article “Enhancing immunity in viral infections, with special emphasis on COVID-19: A review” (Jayawardena et al.). Diabetes Metab. Syndr..

[80] Charitos I.A., Ballini A., Bottalico L., Cantore S., Passarelli P.C., Inchingolo F., D’Addona A., Santacroce L. (2020). Special features of SARS-CoV2 in daily practice. World J. Clin. Cases.

[81] Charitos I.A., Del Prete R., Mosca A., Inchingolo F., Carretta D., Ballini A., Santacroce L. (2020). What We Have Learned for the Future About COVID-19 and Healthcare Management of it?. Acta Biomed..

[82] Cantore S., Ballini A. (2020). Coronavirus Disease 2019 (COVID-19) pandemic burst and its relevant consequences in dental practice. Open Dent. J..

[83] Pham V.H., Gargiulo I.C., Nguyen K.C.D., Le S.H., Tran D.K., Nguyen Q.V., Pham H.T., Aityan S., Pham S.T., Cantore S. (2020). Rapid and sensitive diagnostic procedure for multiple detection of pandemic Coronaviridae family members SARS-CoV-2, SARS-CoV, MERS-CoV and HCoV: A translational research and cooperation between the Phan Chau Trinh University in Vietnam and University of Bari “Aldo Moro” in Italy. Eur. Rev. Med. Pharmacol. Sci..

[84] Santacroce L., Charitos I.A., Bottalico L. (2019). A successful history: Probiotics and their potential as antimicrobials. Expert Rev. Anti-Infect. Ther..

[85] Isacco C.G., Ballini A., De Vito D., Nguyen K.C.D., Cantore S., Bottalico L., Quagliuolo L., Boccellino M., Di Domenico M., Santacroce L. (2020). Rebalance the oral microbiota as efficacy tool in endocrine, metabolic, and immune disorders. Endocr. Metab. Immune Disord. Drug Targets.

[86] Ballini A., Cantore S., Scacco S., Coletti D., Tatullo M. (2018). Mesenchymal stem cells as promoters, enhancers, and playmakers of the translational regenerative medicine 2018. Stem Cells Int..

[87] Ballini A., Gnoni A., De Vito D., Dipalma G., Cantore S., Gargiulo I.C., Saini R., Santacroce L., Topi S., Scarano A. (2019). Effect of probiotics on the occurrence of nutrition absorption capacities in healthy children: A randomized double-blinded placebo-controlled pilot study. Eur. Rev. Med. Pharmacol. Sci..

[88] Inchingolo F., Dipalma G., Cirulli N., Cantore S., Saini R.S., Altini V., Santacroce L., Ballini A., Saini R. (2018). Microbiological results of improvement in periodontal condition by administration of oral probiotics. J. Biol. Regul. Homeost. Agents.

[89] Ballini A., Santacroce L., Cantore S., Bottalico L., Dipalma G., Vito D., Saini R., Inchingolo F. (2018). Probiotics Improve Urogenital Health in Women. Maced. J. Med. Sci..

